# Role of posterior-anterior vertebral mobilization versus thermotherapy in non specific lower back pain

**DOI:** 10.12669/pjms.342.12402

**Published:** 2018

**Authors:** Aftab Ahmed Mirza Baig, Syed Imran Ahmed, Syed Shahzad Ali, Asim Rahmani, Faizan Siddiqui

**Affiliations:** 1 Aftab Ahmed Mirza Baig, Lecturer of Physiotherapy, Department of Physiotherapy, Institute of Physical Medicine & Rehabilitation, Dow University of Health Sciences, Karachi, Pakistan; 2 Syed Imran Ahmed, Assistant Professor of Physiatry, Institute of Physical Medicine & Rehabilitation, Dow University of Health Sciences, Karachi, Pakistan; 3 Syed Shahzad Ali, Assistant Professor of Physiotherapy, Department of Physiotherapy, Institute of Physical Medicine & Rehabilitation, Dow University of Health Sciences, Karachi, Pakistan; 4 Asim Rahmani, Assistant Professor of Neurosurgery, Civil Hospital, Karachi, Pakistan; 5 Faizan Siddiqui, Lecturer of Physiotherapy, Department of Physiotherapy, Institute of Physical Medicine & Rehabilitation, Dow University of Health Sciences, Karachi, Pakistan

**Keywords:** General stretching exercises, Non-specific low back pain, Posterior-anterior mobilization, Thermotherapy

## Abstract

**Background & Objective:**

Low back pain (LBP) is the foremost cause to hamper an individual’s functional activities in Pakistan. Its impact on the quality of life and work routine makes it a major reason for therapeutic consultations. About 90% of the cases with LBP are non-specific. Various options are available for the treatment of LBP. Posterior-anterior vertebral mobilization, a manual therapy technique; and thermotherapy are used in clinical practice, however evidence to gauge their relative efficacy is yet to be synthesised. This study aimed to compare the effectiveness of posterior-anterior vertebral mobilization versus thermotherapy in the management of non-specific low back pain along with general stretching exercises.

**Methods:**

A randomised controlled trial with two-group pretest-posttest design was conducted at IPM&R, Dow University of Health Sciences (DUHS). A total of 60 Non-specific low back pain (NSLBP) patients with ages from 18 to 35 years were inducted through non-probability and purposive sampling technique. Baseline screening was done using an assessment form (Appendix-I). Subjects were allocated into two groups through systematic random sampling. Group-A (experimental group) received posterior-anterior vertebral mobilization with general stretching exercises while group B (control group) received thermotherapy with general stretching exercises. Pain and functional disability were assessed using NPRS and RMDQ respectively. Pre & post treatment scores were documented. A maximum drop-out rate of 20% was assumed. Recorded data were entered into SPSS V-19. Frequency and percentages were calculated for categorical variables. Intragroup and intergroup analyses were done using Wilcoxon signed ranked test and Mann-Whitney Test respectively. A P-value of 0.05 was considered statistically significant.

**Results:**

Pre and post treatment analysis revealed that P-values for both pain and disability were less than 0.05, suggesting significant difference in NPRS and RMDQ scores. Whereas, median scores for both pain and disability were decreased by 75% in experimental group and 50% in control group. For inter group analysis p-values for both pain and disability were found to be less than 0.05.

**Conclusion:**

Both physiotherapeutic interventions, the PAVMs and thermotherapy, have significant effects on NSLBP in terms of relieving pain and improving functional disability. However PAVMs appeared to be more effective than thermotherapy.

## INTRODUCTION

Low back pain (LBP) is increasingly becoming one of the main health concerns nowadays. It affects daily life and work routine and leads to medical consultations.^1^ Formerly considered to be confined only to affluent western countries, the LBP is also becoming a formidable burden to the low and middle-income countries as revealed by the explorations made through the last decade.^2^

The pain or discomfort present arising in the region between costal margin and inferior gluteal folds, with idiopathic cause and devoid of any precise pathology is commonly referred to as Non-specific low back pain.^3^ The lifetime prevalence of LBP is approximately 85% (probably closer to 100% in adults). About 90% of all LBP cases are non-specific low back pain (NSLBP). LBP has attained 31.0% global mean prevalence, 38.0% annual prevalence and 18.3% mean point prevalence. 83.1 million is the recently reported figure of years lived with disability (YLDs) associated with LBP. In Pakistan, LBP is the third leading cause of YLDs and its prevalence has been found to be 19.5 %.^4^ The countries where point prevalence has been reported includes China (34.1%), Bangladesh (20.1%), Iran (14.8), United Kingdom (9.0%) and India (8.4%).^2^

The prevalence of NSLBP is approximately 10 to 25% in young and middle-aged individuals. Even though degenerative changes are minimal in this age group, more physical activities consequently make this group vulnerable to various physical strains. On account of this, incidence of LBP in this age group is high whereas majority of such cases are by and large of NSLBP. Manual workers and labourers who are subjected to heavy physical exertion i.e. weight lifting, repetitive movements, frequent postural vagaries etc. are prone to develop NSLBP.^5^

The clinical practice guidelines (CPGs) developed during the past decades emphasised screening for potentially serious pathologies like infection, precise causes of LBP, and the assessment of pain intensity and functional disabilities. The importance of staying active in patients with NSLBP is now accentuated more than ever. Whereas the use of over-the-counter medications and spinal manual therapy (SMT) like mobilization, manipulation are frequently considered as the first line interventions for symptom control. Supervised exercise, cryotherapy, and thermotherapy and, to a lesser degree, behavioural modification or acupuncture therapies are also recommended.^6^ Among the many treatment options Posterior-Anterior Vertebral Mobilizations (PAVMs) and Thermotherapy are widely used as Physiotherapeutic management, however evidence about their relative efficacy is limited. This study was conducted to explore the effects of the two by comparing the PAVMs with thermotherapy along with General Stretching Exercises (GSEs).

The PAVMs is the joint mobilization technique that is considered as the cornerstone in manual therapy. It can be defined as passive oscillatory movement, which at any point of time during the course, is within the ability of patient to resist. Maitland includes graded Posterior-anterior vertebral mobilizations (PAVMs) as a means of evaluation and treatment of LBP and accompanying stiffness in order to improve function. A careful and comprehensive examination is a re-requisite for the application of the technique.^7^ On the other hand, Thermotherapy is commonly used for the treatment of LBP. It is easy to apply in addition to being cost-effective. Thermotherapy plays putative role in relieving pain and muscle spasm.^8^

## METHODS

A randomized trial was conducted on 60 NSLBP patients those fulfilling inclusion criteria were selected from OPD of neurology Civil Hospital and IPM&R, DUHS, Karachi, Pakistan with non-probability purposive sampling. Inclusion criteria included males and females with age 18-35 years and NSLBP for <3 months. Whereas exclusion criteria included LBP due to specific pathology, patients with neurological deficits in lower limbs with decreased power in myotomes or decreased sensation in dermatomes, patients with neurological diseases (like Stroke, Parkinsonism), any clinical condition that contra indicated mobilization, history of spinal surgery and patients administered epidural injections. Informed consent was taken from all the study participants then they were randomly assigned to either of groups of two with simple random sampling. Each group was having 30 participants, The Group-A received program of PAVMs including, posterior anterior oscillatory movements on specific lumber segments that demonstrate restricted movements and in which pain is reproduced upon graded movement. Three bouts of 30 oscillations were applied at the rate of approximately three oscillations per second.^9^ The Group-B received a program of thermotherapy using hot pack for 20 minutes in prone lying position.^10^ Additionally both groups received program of GSEs as supportive treatment including, stretching exercises for lower back, hamstring & tensor fascia latae for 30 seconds hold for two sets with 10 rep/set.^11^ Outcomes were measured in all patients at the first session and at the last treatment session by using numerical pain rating scale (NPRS) and Rolland Morris disability questionnaire (RMDQ) which were translated into Urdu language for patients’ comprehension. All patients who had difficulty in self-administration of RMDQ due to limited literacy were helped by their caregiver or by person who was not aware to the purpose of RMDQ.

Each subject received total 12 treatment sessions with the frequency of three sessions per week for four consecutive weeks. The duration of each session given to group A (PAVMs +GSEs) and group B (Thermotherapy + GSEs) was about 30 minutes. The Institutional Review Board of Dow University of Health Sciences approval were taken in advanced for this research study (reference no. IRB-432/DUHS/-14).

### Outcome Tools

#### Numerical Pain Rating Scale

The use of NPRS as a self-administered outcome tool is evident by literature as well as clinically with 69% sensitivity and 78% specificity. Patients were requested to fill the pre and post treatment questionnaires. They were allowed to scale their pain severity on NPRS ranging from 0-10 where 0 signifies, No pain. Whereas, 10 signifies most excruciating pain.^12^

### Rolland Morris Questionnaire

The CPGs recommend RMDQ as the authenticated self-administered questionnaires for LBP. It is suitable to recognize the patient present condition comparative to pain, functional disability for assessing any change during treatment. In this greater levels of disability are reflected by higher numbers on a 24-point scale. The RMDQ asks patient to mark whether each of the 24 items is probable to do. The activities are directed by the stem, “Because of my back pain,” thus letting it to be of definite region. The RMDQ has exceptional psychometrics, easy to use, and has been shown to be responsive in RCTs 12. Subjects were asked to rate their score out of 5 grades of severity that is, no pain, little pain, moderate pain, severe pain and intolerable pain.

### Data Analysis

Data were analyzed using SPSS 19 version. Frequency and percentages were shown for categorical variables. Non-parametric tests were employed because study data does not follow the normal distribution. Wilcoxon signed ranked test was used for pre and post treatment evaluation and Mann-Whitney Test was used for differences between two study groups. The value of 0.05 was set as threshold for showing significant association.

**Table-I:** Mean and standard deviation of subjects’ age between experimental and control group.

Study Group	N	Mean Age	Standard Deviation
Experimental	30	26.7	4.921
Control	30	25.76	4.477

## RESULTS

Demographics showed that total 60 patients in this research study with 30 in each group. In both study groups there were 60% male and 40% female with age and gender matching. In descriptive statistics, the Intra group and inter group analysis is determined by drawing different graphs. In intra group analysis, the pre and post treatment NPRS and RMDQ in posterior anterior vertebral mobilization group showed that after the treatment both median pain and disability was decreased by one third. Whereas, the pre and post treatment NPRS and RMDQ in thermotherapy group showed median pain and disability was decreased by half. The Wilcoxon signed ranked test was employed to identify any difference in NPRS and RMDQ scores for pre and post treatment with in each group. Both p-values of pain and disability were less than 0.05 suggesting significant difference in NPRS and RMDQ scores between pre and post treatment for both group. The inter group analysis comprises mean, standard deviation and p-values of the different parameters, showing the improvement between the two groups by the respective treatment procedures.

It was hypothesized that the PAVMs is more effective as compared to thermotherapy in the management of NSLBP along with GSEs. To test this hypothesis the Mann-Whitney Test were applied that results p-values of both NPRS and RMDQ scores, less than 0.05 which was significant and hence enough to reject null hypothesis and accept alternative hypothesis that posterior-anterior vertebral mobilization is more effective as compare to thermotherapy in the management of NSLBP.

## DISCUSSION

NSLBP has been recognised as a common public health problem.^2^ An RCT was conducted to evaluate the effectiveness of PAVMs and thermotherapy along with GSEs in the management of NSLBP. This study included 60 male and female patients with NSLBP and with no serious pathology or any contraindication for the interventions in question. In this study, both the groups evinced significant decreases in NPRS and RMDQ scores at the end of treatment sessions whereas the decrease in NPRS was more pronounced in the experimental group (PAVMs) as compared to the control group (thermotherapy). The significant relief of pain was noted after 12 sessions. When intra-group median values of NPRS and RMDQ were analysed, statistically significant differences in pre and post treatment scores at the end of twelfth session were found for both the groups. In intergroup comparison, statistically significant difference was found between the two groups in relieving pain and functional disability. PAVMs in concert with GSEs proved superior to thermotherapy with GSEs. No study has as yet compared these two techniques, however the present study findings of reduction in pain intensity with application of PAVMs, are consistent with the findings of the studies conducted by Hanrahan et al and Sakulsriprasert et al, indicating PAVMS is effective for pain relief in NLBP.^9^,^13^ It is important however to note that all the participants were given GSEs within joint range as a common conventional method. Bone and muscle are both dynamic structures that respond positively to exercises and adversely to disuse. The loss of muscle mass due to disuse can be substantially reversed by exercise training program. It has hence been suggested that physiotherapists have a responsibility to include GSEs as an essential component of prophylaxis and treatment, in addition to other passive modalities such as massage, mobilisation, manipulation and traction. Ohtsuki et al. in a study describes that GSEs for managing LBP should include back, hamstring and tensor fascia latae stretching within pain free range.^11^

**Table-II:** Post treatment evaluation of pain and disability in Posterior anterior vertebral mobilization group (Experimental) and Thermotherapy group (control).

Variables	PAVMs group Median (Range)	Thermotherapy group Median (Range)	P-value
Pain	2 (6)	3 (4)	0.006*
Disability	4 (14)	6 (10)	0.0115^*^

P-values are obtained by using Mann-Whitney Test

* P-values < 0.05 are considered as significant.

Numerous studies have shown that spinal mobilisation increases pain tolerance and threshold. One mechanism underlying the effects of spinal mobilisation is the ability to alter central sensory processing by removing sub-threshold mechanical or chemical pain stimuli from para spinal tissues. Substantial evidence demonstrates that spinal manipulation/mobilisation elicits paraspinal muscle reflexes and alters motor neurone excitability. The resultant effect of manipulation/mobilisation on these somatic reflexes involves a complex excitation and inhibition neuronal circuitry. The mechanisms behind the effects of vertebral mobilization on these reflexes are beginning to unfold.^7^, ^14^

Christopher et al conducted an RCT with enrolment of 30 participants having NSLBP, 15 in each group. First group was given PAVMs and the second group was treated by press-up exercise. A major reduction in the average pain intensity for both the groups was observed however that was a single session study with a small sample size.^15^ The current study supported the effects of PAVMs on pain intensity. Prasert Sakulsriprasert et al. conducted an RCT in 2010. Improvements in pain intensity, active range of motion, and functional disability were observed in patients with acute NSLBP after receiving either physical therapy or physical therapy combined with spinal mobilization. The study showed no additive effects of spinal mobilisation.^13^ On the contrary this was not the case as revealed by the present study findings and indubitable evidence related to additional effects of spinal mobilisation along with exercise was found.

Kent and fellows in 2010 reported four RCTs in their systematic review on vertebral mobilisation. They stated significant effects in LBP but with too small a sample.^16^ However this study generated evidence that there are significant effects of PAVMs as a specific technique of vertebral mobilisation. Mulkern and colleagues in a Cochrane review of 5 RCTs reported better effects of heat wrap application in reducing pain in NSLBP when compared with non-heat wrap application. All the outcomes were on the basis of short term effects. Authors cumulatively concluded that thermotherapy has some therapeutic effects in comparison to other treatment options.^17^ The current study finding of having significant effects of thermotherapy is in conformity to that study however having proved to be better than thermotherapy this study has unveiled another more prospective avenue of PAVMs for the treatment of NSLBP. French et al in 2006 concluded in a review, that the heat wrap therapy significantly reduced pain after five days compared to oral placebo in two trials on acute and sub-acute LBP. They also reported a trial of 100 participants in which heat wrap along with exercise showed long term pain reduction.^8^ The current study corroborated the evidence that heat therapy with exercise, particularly GSEs is beneficial in acute and sub-acute cases of LBP. Middelkoopet et al. in a systematic review (2011) highlighted a controversy that existed regarding the clinical effects of thermotherapy or superficial heat therapy.^18^ This study affirms significant effects of PAVMs than thermotherapy along with GSEs in NSLBP. Aside from this comparison, both the interventions had significant effects and remained beneficial in producing marked improvement in pain intensity and functional disability.

NSLBP being responsible for majority of the LBP cases, is a major health care problem which poses a huge disease burden on the society. Simple, safe and cost-effective therapeutic procedures such as PAVMs and thermotherapy combined with other main stay or conventional modes of treatments such as exercise like GSEs could prove to be of great value.

### Limitations

Medication acted as confounder as ethical board directed not to alter the prescribed course if any of the study participants is already taking it.

- Limited inclusion criteria were used in the present study that limits generalising the results to the whole target population.
- Long term benefits of PAVMs or thermotherapy in management of NSLBP are not addressed.

## CONCLUSION

In conclusion, the present RCT provides evidence that physiotherapy techniques i.e. PAVMs and thermotherapy have significant effects in relieving pain and improving functional disability in patients with NSLBP. However PAVMs are shown to be more effective in comparison to thermotherapy. Present study highlights that PAVMs along with GSEs should be used in the patients with NSLBP. Moreover, further research is needed to explore their long term role and effectiveness in contrast to other physiotherapy treatments.

### Authors’ Contributions

**AAMB**: Data Collection and manuscript writing.

**AR and FS**: Data collection.

**SSA:** Review and editing of manuscript.

**SIA**: Review and final approval of manuscript.
